# Inhibition of extranodal NK/T-cell lymphoma by Chiauranib through an AIF-dependent pathway and its synergy with L-asparaginase

**DOI:** 10.1038/s41419-023-05833-w

**Published:** 2023-05-09

**Authors:** Tianxiao Gao, Jieye Huang, Haofan Yin, Jiajia Huang, Jinye Xie, Ti Zhou, Wei Fan, Xia Yang, Guoquan Gao, Zhiming Li

**Affiliations:** 1 Department of Nuclear Medicine, Sun Yat-sen University Cancer Center, State Key Laboratory of Oncology in South China, Collaborative Innovation Center for Cancer Medicine, Guangzhou, 510060 P.R. China; 2 Department of Medical Oncology, Sun Yat-sen University Cancer Center, State Key Laboratory of Oncology in South China, Collaborative Innovation Center for Cancer Medicine, Guangzhou, 510060 P.R. China; 3 Department of Biochemistry, Zhongshan School of Medicine, SunYat-sen University, Guangzhou, China; 4 Guangdong Engineering & Technology Research Center for Gene Manipulation and Biomacromolecular Products, Sun Yat-sen University, Guangzhou, China; 5 Guangdong Provincial Key Laboratory of Brain Function and Disease, Sun Yat-sen University, Guangzhou, China; 6 Key Laboratory of Tropical Disease Control (Sun Yat-sen University), Ministry of Education, Guangzhou, China

**Keywords:** Cancer therapy, Preclinical research

## Abstract

Extranodal NK/T-cell lymphoma (NKTL) is a rare and aggressive form of extranodal lymphoma with a poor prognosis. Currently, there are very limited treatment options for patients with advanced-stage disease or those with relapsed/recurrent disease. Here we show that Chiauranib, an orally small molecule inhibitor of select serine-threonine kinases (aurora B, VEGFRs, PDGFR, CSF1R, c-Kit), inhibited NKTL cell proliferation, induced cell cycle arrest, as well as suppressed the microvessel density in vitro and in vivo similar as in other types of cancer cells. Surprisingly, Chiauranib unfolded a new effect to induce apoptosis of NKTL cells by triggering AIF-dependent apoptosis other than the traditional cyt-c/caspase mitochondrial apoptosis pathway. The knockdown of AIF in vitro and in vivo dramatically blocked the efficacy of Chiauranib on NKTL. Mechanistically, the release of AIF from mitochondria is due to the upregulation of VDAC1 by the AKT-GSK3β pathway and activation of calcium-dependent m-calpain, which promotes the cleavage of VDAC1 and therefore permits the release of AIF. Notably, the low expression of Bax in both NKTL cells and patient tissues restrained the cyt-c release. It resulted in the inhibition of cyt-c/caspase mitochondrial pathway, suggesting that drugs targeting this traditional pathway may not be effective in NKTL. Furthermore, we found that L-asparaginase triggered CD95 (Fas/Apo-1)-caspase 8-caspase 3 apoptotic pathway in NKTL cells, and combination of Chiauranib and L-asparaginase exhibited a synergistic effect, suggesting a feasibility to combine these two drugs for effective treatment of NKTL. This study demonstrates Chiauranib’s positive efficacy toward NKTL through the activation of the AIF-dependent apoptosis pathway for the first time. The novel and multi-targets of Chiauranib and the synergistic effect with L-asparaginase may provide a promising therapy for NKTL patients.

## Introduction

Extranodal NK/T-cell lymphoma (NKTL), nasal type (ENKL), is a typical kind of extranodal lymphoma characterized by vascular damage and destruction, prominent necrosis, cytotoxic phenotype, and association with EBV. China, Japan, South Korea, and parts of South America are the main predilection areas [1].

NKTL is an aggressive type of lymphoma with a poor prognosis. Up to now, still few phase III clinical trials can help guide therapy. Even fewer data are found in the relapsed/refractory cohort of patients. NKTL was proven insensitive to CHOP (cyclophosphamide, doxorubicin, vincristine, and prednisone) chemotherapy, even at the maximal dose intensity [2, 3]. Lately, Asp (asparaginase) [4, 5] or Peg-Asp (pegylated asparaginase) showed improved efficiency in NKTL. Regimens such as P-GEMOX (pegaspargase, gemcitabine, and oxaliplatin) are now prevalent in clinical applications [6–9]. Although approximately 70–90% of early-stage and 15–65% of advanced-stage patients achieve complete remission (CR) after primary therapy, a proportion of patients eventually experience a relapse from 25 to 60%, depending on the treatment modality [10]. Currently, the long-term survival rate of patients with advanced NKTL is still low. Thus, new drugs and practical therapeutic approaches are urgently needed.

Compared to other peripheral T-cell lymphomas or normal NK cells, genes related to angiogenesis were overexpressed in NKTL [11–13]. We also found in our clinical patients that the combination of Avastin with chemotherapeutics reduced the recurrence rate and prolonged patients’ survival rate, indicating that targets related to angiogenesis are crucial in NKTL [14]. The PDGFR signaling pathway was also found activated in NKTL cells [11]. C-KIT mutations have been confirmed in NKTL patients in many countries, although the proportion of patients with mutations varies [15–17].

Chiauranib is an orally available small molecule inhibitor that inhibits selected serine-threonine kinases, including aurora kinase B (aurora B), vascular endothelial growth factor receptors (VEGFRs), platelet-derived growth factor receptor (PDGFR), colony-stimulating factor-1 receptor (CSF1R) and Tyrosine-protein kinase KIT (c-Kit) [18]. At present in China, phase III clinical trials involving ovarian cancer (NCT04921527) and small-cell lung cancer (NCT04830813) are currently under investigation, and Phase II clinical trials for non-Hodgkin’s lymphoma (NCT03974243), liver cancer (NCT03245190) and triple-negative breast cancer (NCT05336721) are in progress. In the United States, phase 1b/2 clinical trials are running concurrently for small-cell lung cancer (NCT05271292).

Considering the targets of Chiauranib on tumor proliferation and angiogenesis closely related to NKTL and the progress in clinical trials, Chiauranib was selected to explore its potential effects and underlying mechanism on NKTL in the present study. Here, we demonstrate the potent effects of Chiauranib on NKTL with a unique mechanism besides the known targets and hope to provide insight into the treatment of specific lymphoma NKTL.

## Results

### Chiauranib exhibits potent antitumor activity against NKTL in vitro and in vivo

**Fig. 1:**
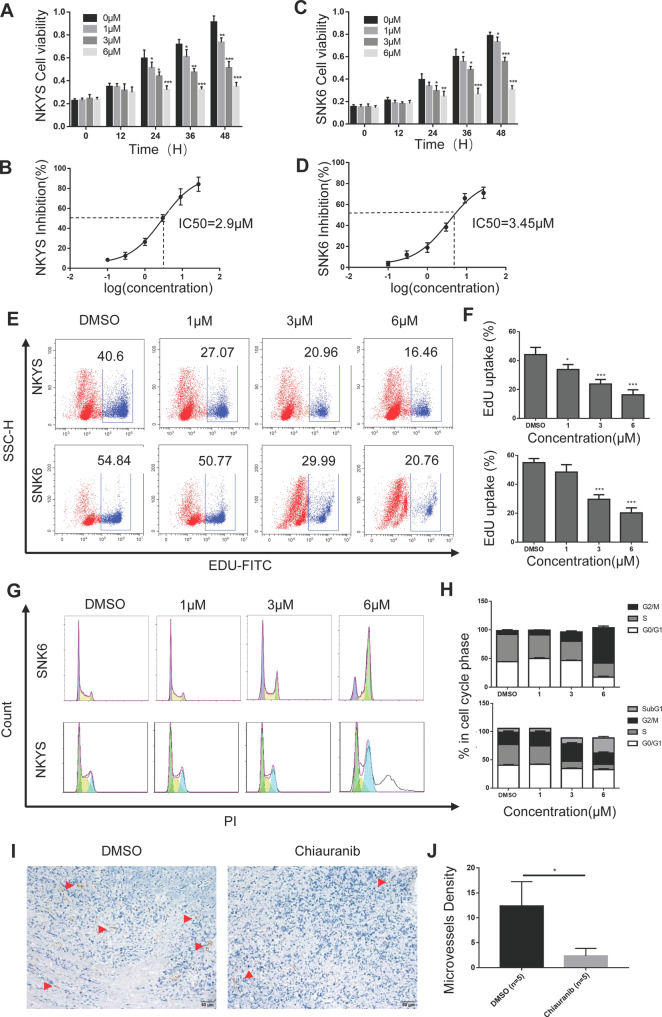
Chiauranib exhibits potent antitumor activity against NKTL in vitro and in vivo. NKYS and SNK6 cell lines were treated with DMSO or various concentrations of Chiauranib for 0, 12 h, 24 h, and 48 h, respectively. CCK8 assay was performed in both **A**, **B** NKYS and **C**, **D** SNK6 cell lines. NKYS and SNK6 cell lines were treated with various concentrations of Chiauranib for 48 h. **E**, **F** The cells were further incorporated with EdU and detected with flow cytometry. **G**, **H** Cell cycle analysis through PI staining was followed by flow cytometry for NKYS and SNK6 cell lines after treatment. **I**, **J** Female 4-week-old NOD/SCID mice was intraperitoneally implanted with SNK6 cells. Three weeks after the inoculation of NKTL, cell tumors were excised and processed for immunostaining using a rat anti-mouse CD31 endothelial marker (shown in red arrow). The MVD of the tumors was determined by counting CD31-positive areas in 10 fields/serial tumor sections from five animals per group (shown in red arrow).

To determine whether NKTL is sensitive to Chiauranib, we tested this drug on two NKTL cell lines, NKYS and SNK6. Significantly reduced cell viability was observed in both cell lines, which relies on the time point and concentration of Chiauranib (Fig. 1A–D). IC50 of Chiauranib was 2.9 μM and 3.45 μM on NKYS and SNK6, respectively. Dose-dependent EdU uptake was also shown in both cell lines, indicating inhibition of cell proliferation (Fig. 1E, F). Flow cytometry analysis verified that Chiauranib induced the pronounced cell cycle arrest in the G2/M and Sub G1 phase, in conformity with the anti-mitotic effect of blocking Aurora B (Fig. 1G, H). In vivo experiments with NOD/SCID mice showed the anti-angiogenesis function by reducing microvessel density within NKTL tissues (Fig. 1I, J).

### Chiauranib induces apoptosis in NKTL cell lines and NKTL xenograft mice

**Fig. 2:**
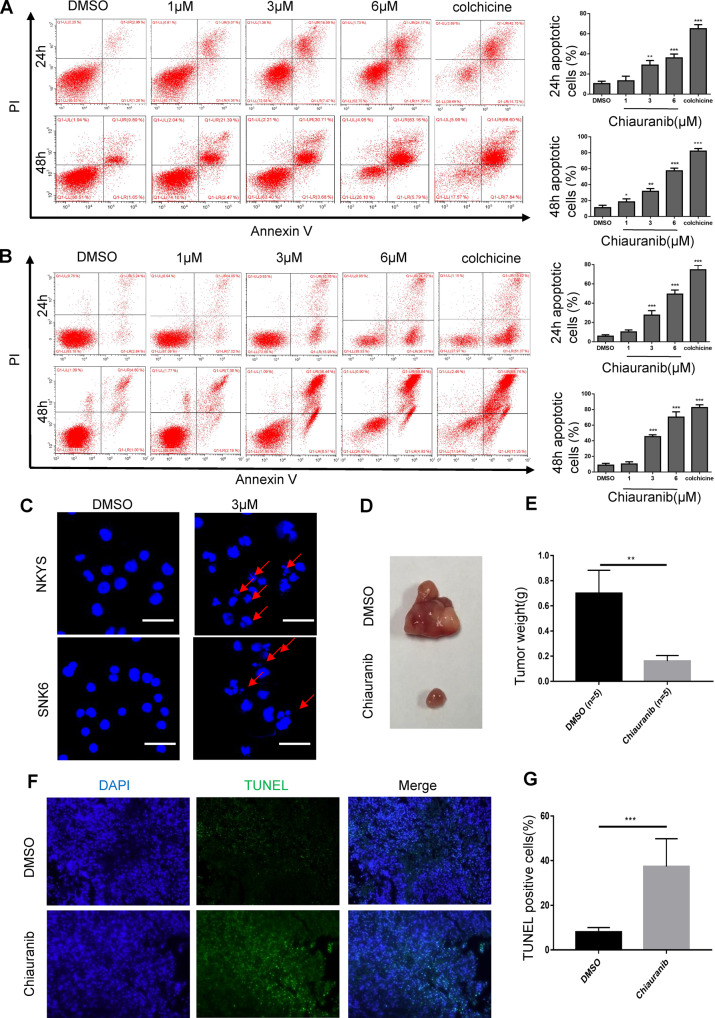
Chiauranib induces apoptosis in NKTL cell lines and NKTL xenograft mice. Annexin V-PI staining of **A** NKYS and **B** SNK6 cells exposed to Chiauranib. Cells were treated with Chiauranib, Colchicine (5 μM), and DMSO for 24 h and 48 h, respectively, before staining. **C** Hoechst staining of NKYS and SNK6 cells treated by DMSO or Chiauranib for 48 h, nucleus fragmentations were as shown. Bar = 50 µm. **D**, **E** Representative pictures of tumor tissues and tumor weight of NKTL xenograft mice were measured 3 weeks after inoculation (*n* = 5 per group). **F**, **G** Tunnel assay of mice tumor tissue was performed. Bar = 50 µm.

During the above experiments on SNK6 and NKYS cells, we unexpectedly observed cell morphology change after treatment of Chiauranib, including irregular shape and shrinkage of the cell, which led us to question if Chiauranib was able to trigger the apoptosis pathway in addition to the functions of designed targets. Annexin V-PI staining of NKYS and SNK6 was analyzed with flow cytometry after Chiauranib treatment with different concentrations and time points (Fig. 2A, B). Hoechst staining also indicates apoptosis processing as nucleus fragmentations were seen after treatment (Fig. 2C). We predict that Chiauranib also affects the apoptosis pathway in NKTL cells since the phenotype cannot be explained according to the previously revealed mechanism of Chiauranib. Further on, we tried to verify this finding in vivo. Chiauranib inhibited the growth of tumors in SNK6-injected mice (Fig. 2D, E). TUNEL experiment was performed with tumor section, which further confirmed that Chiauranib induces apoptosis in mice tumor model (Fig. 2F, G). Thus, in vivo experiment results are consistent with results shown in vitro. In conclusion, Chiauranib induces apoptosis in NKTL and eliminates the growth of tumor cells.

### Apoptosis induced by Chiauranib in NKTL cell lines is not promoted by the classical caspase-dependent apoptosis pathway

**Fig. 3:**
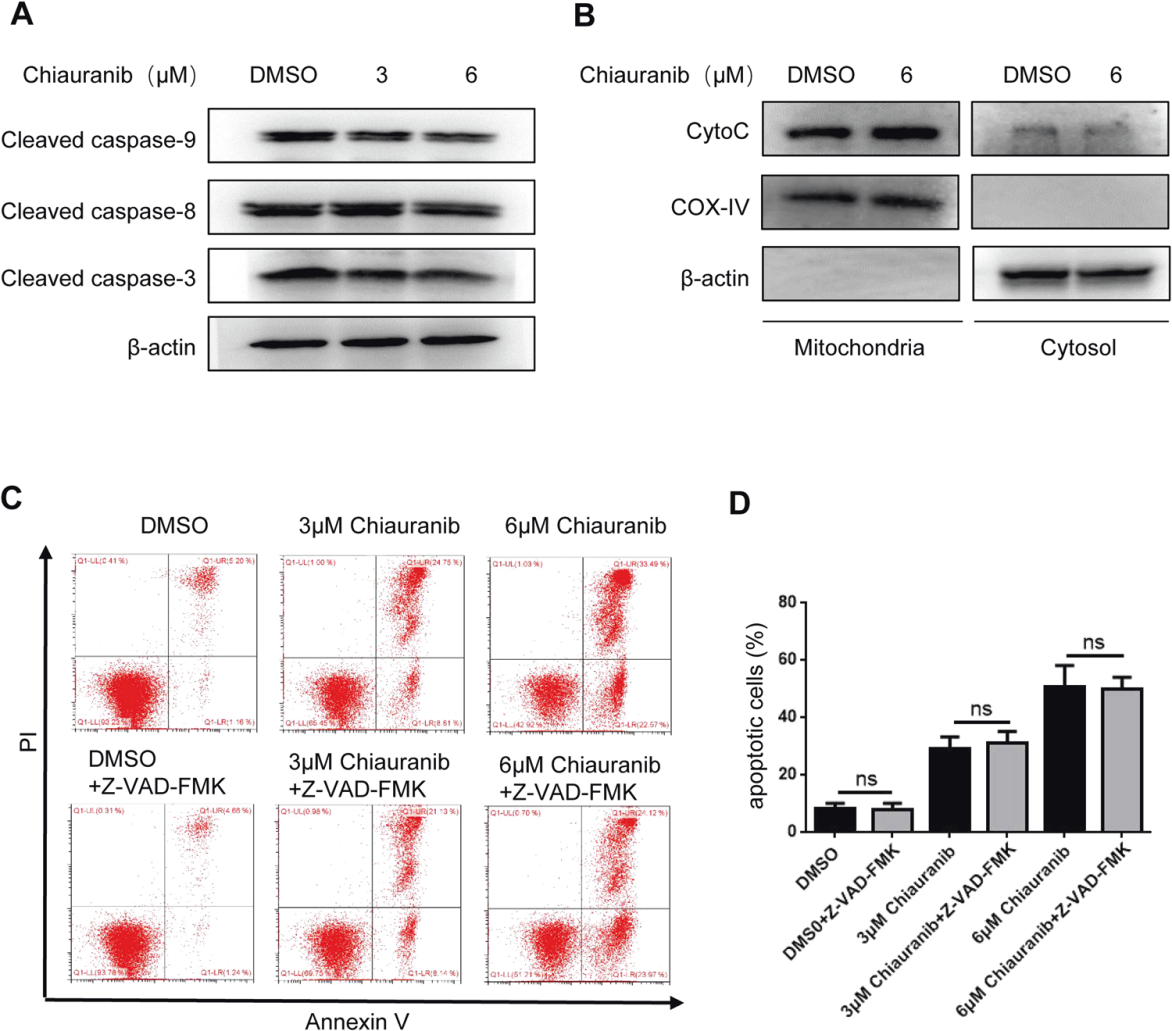
Apoptosis induced by Chiauranib in NKTL cell lines was not promoted by the classical mitochondrial apoptosis pathway. **A** Protein extracts from SNK6 cells were analyzed by immunoblotting for cleaved caspase 8, 9, 3, and β-actin. SNK6 cells were treated with DMSO or Chiauranib for 24 h. **B** Immunoblotting analysis of mitochondria and cytosol enriched fractions of SNK6 cells treated with Chiauranib or DMSO for 24 h. **C**, **D** Annexin V-PI staining of SNK6 cells treated by DMSO or Chiauranib with or without Z-VAD-FMK (50 μM).

To prove the possible apoptosis pathway, we first observed the classical apoptotic caspases, including caspase-3, -9, and -8. However, results of immunoblotting showed a comparable level of cleaved caspases after Chiauranib treatment in the SNK6 cell line (Fig. 3A). We further isolated mitochondria and cytosol protein to detect the release of cytochrome c (cyt-C) from mitochondria, which is the major upstream of the pathway. Results proved that cyt-C was also comparable in both lysates (Fig. 3B). To confirm, we treated cells with Z-VAD-FMK, an irreversible pan-caspase inhibitor. Z-VAD-FMK failed to rescue apoptosis by Chiauranib, further proving apoptosis is caspase-independent and may be activated by another pathway (Fig. 3C, D).

### Chiauranib induces apoptosis by regulating the Apoptosis-inducing factor (AIF) in NKTL cells

**Fig. 4:**
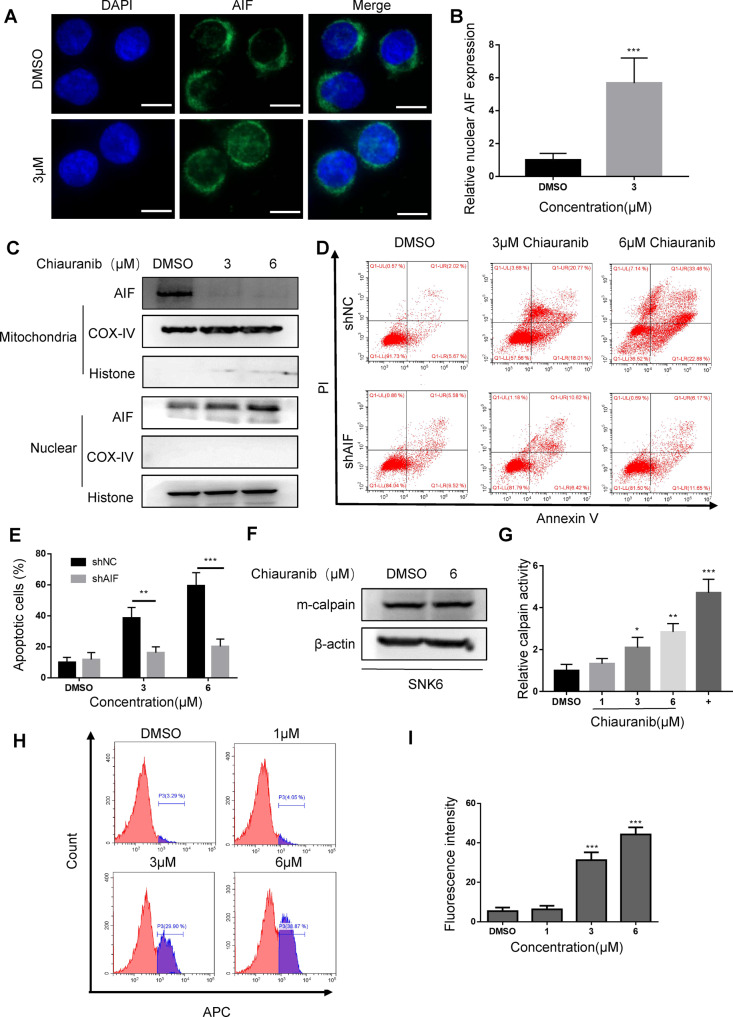
Chiauranib induces apoptosis by regulating the Apoptosis-inducing factor (AIF) in NKTL cell lines. **A**, **B** Immunofluorescence staining for AIF (green) and DAPI (blue) of SNK6 cells treated with Chiauranib or DMSO 24 h. Bar = 50 µm. **C** Immunoblotting analysis of mitochondria and nucleus enriched fractions of SNK6 cells treated with Chiauranib or DMSO for 24 h. COX-IV and Histone serve as loading controls for mitochondrial and cytosolic fractions, respectively. **D**, **E** Annexin V-PI staining of SNK6 cells transduced with control shRNA or shRNA targeting AIF. Cells were exposed to Chiauranib or DMSO for 24 h before staining. Efficacy of shRNA is shown in Supplementary Fig. 1A. **F** Immunoblotting of SNK6 cells treated with Chiauranib or DMSO for detecting the total amount of m-calpain in SNK6 cells. **G** Activity of m-calpain was detected by Fluorescence calpain activity assay after treatment of Chiauranib or DMSO. The positive control group is labeled as “+”. **H**, **I** Flow cytometry detection of Fluo-3 was used to observe the calcium activity after Chiauranib or DMSO treatment in SNK6 cells.

AIF is a caspase-independent protein that is induced under cell stress and leads to DNA fragmentation and chromatin condensation. This procedure starts when the AIF precursor is cleaved into a mature form and anchors the mitochondria membrane. When apoptosis stimulation triggers the cell, mature AIF is released from mitochondria to cytosol and then finally enters nucleus, interacting with endonuclease and leading to chromatin condensation and DNA fragmentation, causing cell apoptosis [19–21]. We hypothesized that Chiauranib triggers AIF activation and leads to cell apoptosis in NKTL cells without involving caspase-dependent signaling. After Chiauranib treatment, AIF was accumulated in the nucleus (Fig. 4A, B). Immunoblotting also shows the decreasing protein level in the mitochondria and inducing necleus translocation (Fig. 4C). Due to these results, we knocked down AIF in SNK6 cells, and AIF blockage rescued the apoptosis induced by Chiauranib (Fig. 4D, E). The release of AIF was known to be involved with mitochondria m-calpain. Mitochondrial m-calpain truncates VDAC in a Ca^2+^-dependent manner. This cleavage of VDAC changes the structure of VDAC and promotes the release of AIF from mitochondria [22]. In the SNK6 cells, the total amount of m-calpain was comparable (Fig. 4F). However, the activity of m-calpain (Fig. 4G) and Ca^2+^ concentration (Fig. 4H, I) after Chiauranib treatment was significantly activated. Thus, the AIF release and necleus translocation are responsible for the apoptosis in NKTL cells.

### Chiauranib promotes AIF release into the nucleus via the AKT-GSK3B-VDAC pathway

**Fig. 5:**
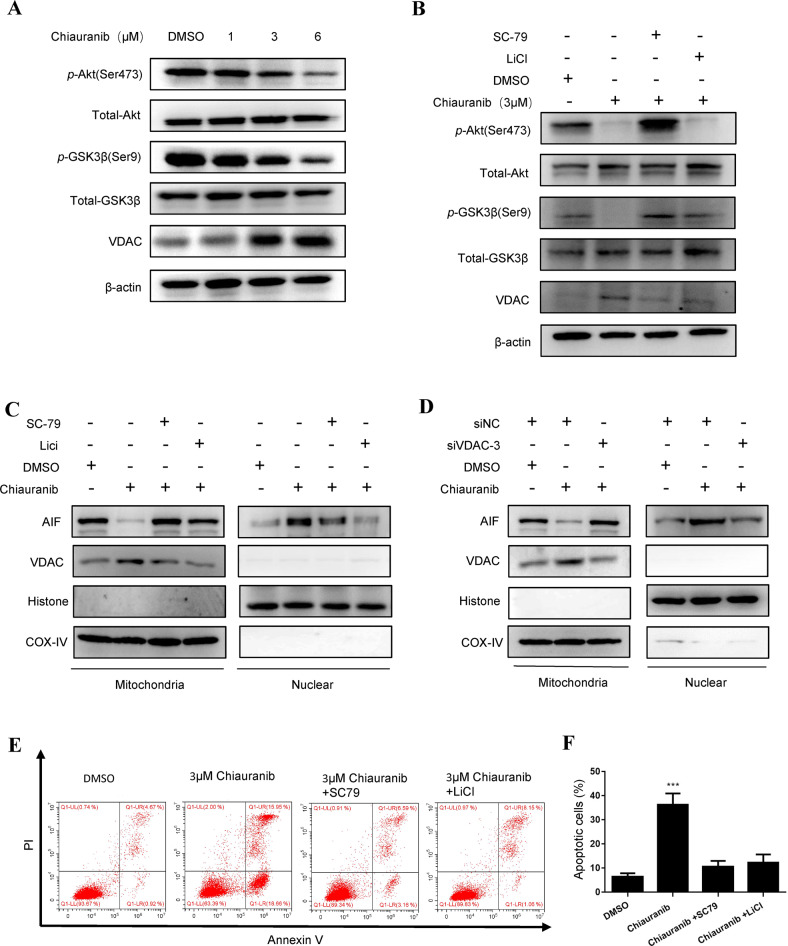
Chiauranib promotes AIF release into the nucleus via the AKT-GSK3B-VDAC pathway, thereby inducing apoptosis. **A**–**D** SNK6 cells were treated with Chiauranib or DMSO for 24 h. SNK6 were pretreated with 5 µM SC-79 or 5 mM LiCl for 30 min before Chiauranib treatment. Immunoblotting analysis of the AKT-GSK3B-VDAC pathway was performed. **C**, **D** Immunoblotting analysis of mitochondria and nucleus enriched fractions of SNK6 cells. siNC (control) and siVDAC-3 was transfected into SNK6 before experiment. The efficacy of siRNA is shown in Supplementary Fig. 1B. **E**, **F** Annexin V-PI staining of SNK6 cells treated with Chiauranib or DMSO for 24 h with or without SC-79, LiCl.

VDAC functions as an opener of the mitochondrial permeability transition pore (mPTP), which releases AIF, cyt-C, etc., into the cytoplasm and further activates the apoptosis pathway [23]. AKT-GSK3β signal pathway is related to the stability and location of VDAC1. This inactivates AKT and stimulates the activity of GSK3β, which promotes the phosphorylation of VDAC1 (p-VDAC1). p-VDAC1 blocks the ubiquitin-dependent degradation of VDAC1 and facilitates its accumulation. Subsequently, the upregulated VDAC1 partially translocates onto the mitochondrial membrane and initiates the release of molecules and cell apoptosis [24, 25]. We therefore detected the AKT-GSK3β pathway and VDAC1 in our cell line after Chiauranib treatment. Phos-GSK3β (Ser-9), the non-active form of GSK3β is reduced by the decrease of phos-AKT (Ser-473), therefore stabilizing VDAC1 (Fig. 5A). The Akt activator SC-79 and GSK3β inhibitor LiCl can respectively reduce the content of VDAC1 in total cell lysates (Fig. 5B). SC-79 and LiCl also regulated AIF release from mitochondria and nucleus translocation (Fig. 5C), which links us to the relationship between the AKT-GSK3β pathway and AIF. Further on, directly knocking down VDAC1 in the SNK6 cell line also leads to decreased release of AIF (Fig. 5D). Moreover, the regulation of the AKT-GSK3β pathway by SC-79 and LiCl was able to rescue the apoptosis in the SNK6 cell line (Fig. 5E, F). From the above, after Chiauranib treatment in the SNK6 cell line, the AKT-GSK3β pathway promotes the stabilizing of VDAC1, therefore allowing a transition pore for mature AIF to be released from mitochondria and therefore promoting apoptosis.

### NKTL lacks BAX expression, which leads to an inability to induce mitochondrial cyt-C released apoptosis

**Fig. 6:**
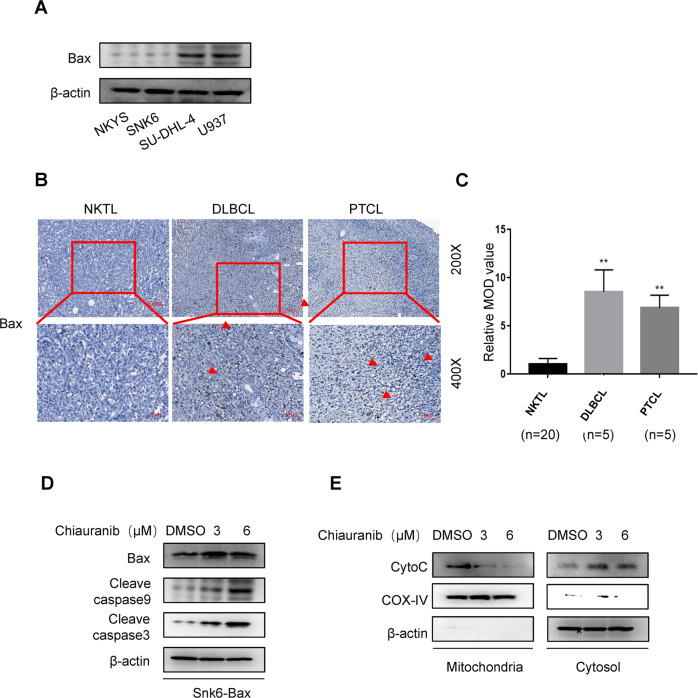
NKTL lacks BAX expression, which leads to the inability to induce mitochondrial cyt-C released apoptosis. **A** Immunoblotting analysis of Bax expression in different types of lymphoma cell lines. **B**, **C** Immunohistochemistry assay of Bax expression in human NKTL, DLBCL, and PTCL tissue (shown in red arrow). *n* for independent patient number. **D** Immunoblotting analysis of caspase pathway in Bax overexpressed SNK6 cells treated with Chiauranib or DMSO. Bax overexpression is shown in Supplementary Fig. 1C. **E** Immunoblotting analysis of mitochondria and cytosol enriched fractions of SNK6 cells after Bax overexpression.

The AKT-GSK3β-VDAC1 pathway is known to contribute to both AIF and cyt-C release. In most cancer cells, when VDAC1 is activated, both types of apoptosis pathways are triggered [26–28]. However, in SNK6 cell lines, the release of cyt-C and downstream caspase signaling changes are unfound. It is likely that in this cell line, cyt-C release is inhibited by other mechanisms. BAX is known to be a key molecular for the cyt-C release and caspase-dependent apoptosis pathway. BAX forms a complex with VDAC1 to extend the pore on mitochondria, allowing cyt-C into the cytoplasm. The BAX channel inhibitors prevent cyt-C release from mitochondria, inhibiting the apoptosis process [29, 30]. We tested the protein level of BAX on two NKTL cell lines, including SNK6 and NKYS. Compared to B lymphocyte cell line SU-DHL-4 and histiocytic lymphoma cell line U937, BAX was low expressed in the NKTL cell lines (Fig. 6A). To further confirm BAX expression, we tested NKTL, Diffuse large B-cell lymphoma (DLBCL), and Peripheral T-cell lymphoma (PTCL) patient samples using immunohistochemical. Similarly, NKTL had a low expression of BAX (Fig. 6B, C). We then overexpressed BAX in the SNK6 cell line and observed the increase of downstream caspase signaling (Fig. 6D) and release of cyt-C (Fig. 6E). When BAX is overexpressed, Chiauranib treatment is able to trigger the caspase-dependent pathway, suggesting that NKTL evades the caspase-dependent apoptosis due to the low expression level of BAX.

### Chiauranib induces AIF-dependent apoptosis and eliminates tumor growth of NKT lymphoma in vivo

**Fig. 7:**
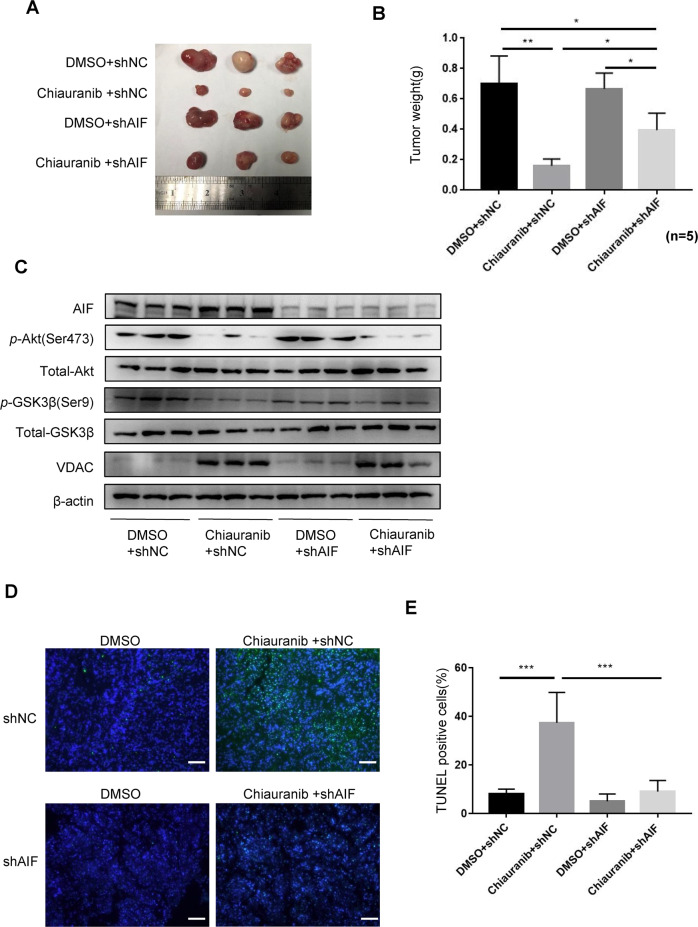
Chiauranib induces AIF-dependent apoptosis and eliminates tumor growth of NKT lymphoma in vivo. Female 4-week-old NOD/SCID mice were randomly divided into four groups, five mice per group. Each group was intraperitoneally implanted with SNK6 cells transduced with control shRNA or shAIF. Human recombinant IL-2 (42 U/g) was intraperitoneal injected every other day. Mice received Chiauranib or DMSO treatment (0.18 μmol/g, intraperitoneal injection, every other day) for 1 week after inoculation with NKTL cells. **A** Representative pictures of tumor tissues from four groups of mice. **B** Tumor weight (g) was measured 3 weeks after the inoculation of NKTL cells after implantation; *n* = 5 per group. **C** Protein extracts from mice tumor tissue were analyzed by immunoblotting for the AKT-GSK3B-VDAC pathway. **D**, **E** Tunnel assay of mice tumor tissue. Bar = 50 µm.

We found the role of Chiauranib in promoting AIF-induced apoptosis on NKTL cell lines. To verify this finding in vivo, we implanted AIF-knockdown SNK6 cells in NOD/SCID mice. Tumor mass indicates that Chiauranib’s function is partially counteracted when injected with shAIF-SNK6 (Fig. 7A, B). Tumor samples were lysed for immunoblotting. The AKT-GSK3β-VDAC1 pathway was activated by Chiauranib, similar to results in vitro (Fig. 7C). TUNEL experiment was performed with the tumor section. Chiauranib lost the ability to induce apoptosis in mice injected with shAIF-SNK6 compared to the group injected with shNC-SNK6 (Fig. 7D, E). In vivo experiment results further confirmed the role of the AIF pathway in Chiauranib treatment.

### Chiauranib and L-asparaginase exhibited a synergistic effect of apoptosis in NKTL

**Fig. 8:**
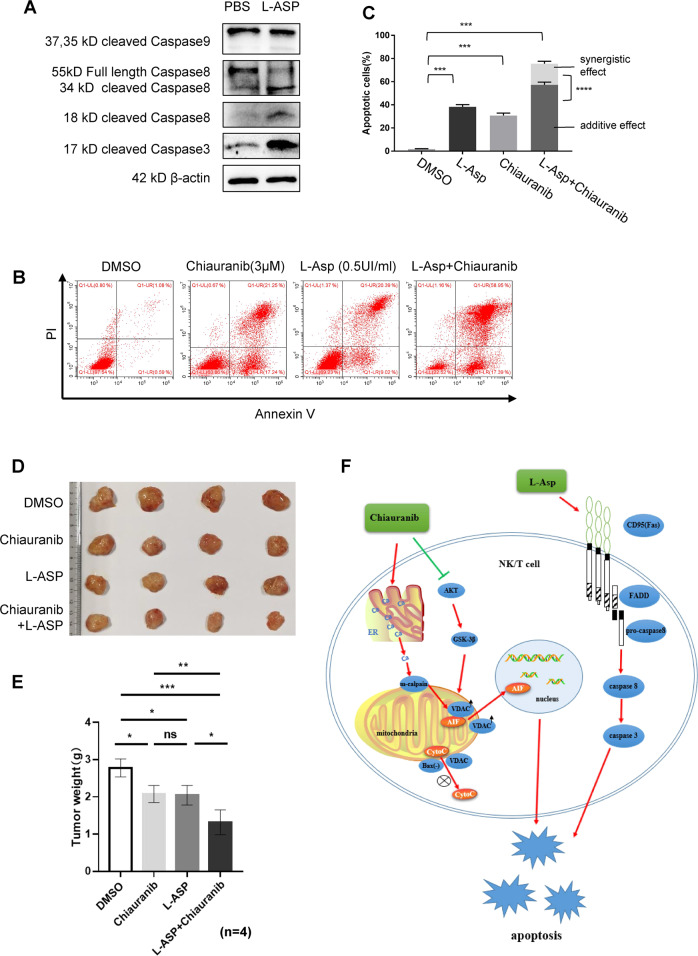
Chiauranib and L-asparaginase exhibited a synergistic effect of apoptosis in NKTL. **A** Immunoblotting analysis of SNK6 cells treated with L-asparaginase (0.5 UI/ml). **B**, **C** Annexin V-PI staining of SNK6 cells treated with Chiauranib and L-asparaginase, respectively, and combinate. **D**, **E** Male 4-week-old NOD/SCID mice injected with SNK6 cells were randomized and divided into four groups respectively (*n* = 4 in each group). Two weeks later, mice received DMSO, 330 IU/kg of L-asparaginase, 0.18 μmol/g Chiauranib, or a combination of both every other day. Representative pictures and the weight of the tumor were shown. **F** Chiauranib can eliminate NKTL growth by triggering AIF-dependent apoptosis. The release of AIF from mitochondria is due to (1) mitochondrial m-calpain activation truncates VDAC in a Ca^2+^-dependent manner, and cleavage of VDAC promotes the release of AIF from mitochondria. (2) The AKT-GSK3β signaling pathway was triggered to potentiate VDAC1 phosphorylation, which contributes to the stability of VDAC1, therefore, allowing AIF to enter the nucleus and induce DNA fragmentation and chromatin condensation. Meanwhile, lack of BAX expression in NKTL leads to the inability to induce mitochondrial cyt-C released apoptosis. L-asparaginase triggered CD95(Fas/Apo-1)-caspase 8-caspase 3 apoptotic pathway in NKTL cells, and the combination of Chiauranib and L-asparaginase exhibited a synergistic effect, suggesting the feasibility of combining these two drugs for effective treatment of NKTL.

As mentioned before, the most widely used treatment in clinics nowadays is L-asparaginase, which was reported as CD95 (Fas/Apo-1)-caspase 8-caspase 3 pathway inducer [31]. We verified that L-asparaginase triggered the caspase 8-caspase 3 pathway without affecting caspase 9 (Fig. 8A). Therefore, the diverse mechanism of the two drugs led us to discover their combination of use. We detected the apoptosis rate of the SNK6 cell line treated with Chiauranib and L-asparaginase, respectively, and combinate. Results showed a synergistic effect between Chiauranib and L-asparaginase in inducing apoptosis, as shown (Fig. 8B, C). The actual apoptosis for the combination was 75.58%, greater than the calculated additive effect of 57.29% (Fig. 8C). In vivo, our data also show a reduction of tumor weight in the combined group when compared to the single drug group (Fig. 8D, E).

For further consideration of the clinical application, we looked into an updated molecular subtyping scheme of NKTL. Clinical patients were identified with three molecular subtypes (TSIM, MB, and HEA) with different clinical outcomes. Cell line SNK6 showed transcriptome features of the MB subtype [32]. We further identified the character of NKYS. According to the RT-PCR results, the SNK6 cell line was confirmed as an MB subtype with high expression levels of MGA, MYC, and MEF2C. NKYS showed transcriptome features of the TSIM subtype, with PD-L1/2 overexpression (Supplementary Fig. 2A). Thus, the effects on the two cell lines indicate MB and TSIM subtypes may be sensitive to Chiauranib.

## Discussion

From our paper and other studies, Chiauranib has been demonstrated to be effective in inhibiting the rapid proliferation of tumor cells and tumor angiogenesis, proving its function of achieving antitumor efficacy through designed targets [18, 33]. Our discovery further suggested that Chiauranib can eliminate NKTL not only by aiming the designed targets but also triggering AIF-dependent apoptosis. Knockdown of AIF remarkably rescues apoptosis and attenuates the antitumor efficiency in vivo and in vitro. The release of AIF from mitochondria is due to m-calpain and AKT-GSK3β-VDAC1 pathway activation, therefore allowing AIF to enter the nucleus and induce DNA fragmentation and chromatin condensation. Importantly, this progress of AIF-dependent apoptosis is independent of the cyt-C-caspase apoptosis pathway.

Interestingly, both cyt-C and AIF are known to be downstream of the AKT-GSK3β-VDAC1 pathway. Our study shows a low expression level of BAX in NKTL cell lines and patient samples prevented the release of cyt-C from mitochondrial (Fig. 6), therefore incapable of triggering classical mitochondrial apoptosis, while overexpression of BAX in NKTL cell line can re-stimulate the pathway (Fig. 6D, E). We therefore conclude in NKTL, the AIF pathway can be triggered with the absence of BAX, but the cyt-C pathway relies on the existence of BAX, which might lead us to the guidance of clinical use. As we mentioned before, the poor outcome of the CHOP (cyclophosphamide, doxorubicin, vincristine, and prednisone) regimen in NKTL patients is likely related to this finding. Cyclophosphamide, doxorubicin, and vincristine, three of the main recipes in this program, were all shown to affect tumor cell death by stimulating apoptosis through cleavage of the caspase 3 and caspase 7 or caspase 9, which correlates with the mitochondrial pathway. Expression of BAX was also mentioned to enhance cytotoxicity with cyclophosphamide [34–37]. We put forward the possibility that chemotherapy related to the cyt-C mitochondrial apoptosis pathway, such as CHOP, may be insufficient for treating localized nasal NKTL due to the low expression level of BAX. Chiauranib, on the other hand, can circumvent the problem by triggering another AIF-dependent pathway.

In this study, we also showed a synergistic effect between Chiauranib and L-asparaginase in apoptosis induction. Through our experiment, we identified that L-asparaginase triggers CD95(Fas/Apo-1)-caspase 8-caspase 3 pathway without affecting caspase 9, synergizing with Chiauranib in inducing apoptosis in NKTLs by targeting different apoptotic pathways (Fig. 8A–C). This prospect the future application of Chiauranib in combination with L-asparaginase regimens or as an alternative treatment for L-asparaginase-resistant relapsed/refractory patients.

Genomic alteration-based molecular subtypes of NKTL were shown to be strongly associated with clinical outcomes [32]. Our results revealed the in vitro sensitivity of Chiauranib on cell line SNK6 and NKYS, representing MB and TSIM subtypes, respectively (Supplementary Fig. 2). Therefore, helping us to predict the availability of Chiauranib on MB and TSIM subtypes.

Thus, from the present results, we revealed the potential role of Chiauranib in the treatment of NKTL due to the high coincidence of crucial targets and its unique AIF-apoptosis pathway, as well as the synergistic effect with L-asparaginase. Our finding may provide a promising therapy for NKTL patients.

## Material and methods

### Human samples

Human lymphoma tissue samples were obtained from Sun Yat-Sen Cancer Center (Guangzhou, China). Tissue samples were obtained from living patients undergoing biopsy procedures; fixation and embedding were performed shortly after to store them for further use. The 30 lymphoma patients included 20 NK/T-cell lymphoma (NKTL), 5 diffuse large B-cell lymphomas (DLBCL), and 5 peripheral T-cell lymphomas (PTCL). All procedures were performed under consensus agreements and in accordance with the Chinese Ethical Review Committee.

### Cell lines and culture

The NKTL cell lines NKYS and SNK6 were from Professor Mingzhi Zhang from The First Affiliated Hospital of Zhengzhou University. SU-DHL-4 and U937 cell lines were from Dr. Guoquan Gao from Sun Yat-sen University. All cells were cultured in RPMI 1640 (Gibco, USA) and supplemented with 10% FBS (Gibco, Uruguay), 1% Penicillin-Streptomycin (Hyclone, USA), and 700 U/ml of recombinant human IL-2 at 37 °C in a humidified incubator containing 5% CO_2_.

### Tumor xenograft models

Female 4-week-old NOD/SCID mice (16–20 g) were purchased from Vital River Laboratory Animal Technology Co., Ltd. (Beijing, China). Tumor xenograft models of the stable shNC-SNK6 or shAIF-SNK6 cells (2 × 10^6^) were performed by intraperitoneal injection (*n* = 10 in each cell line). Mice also received human recombinant IL-2 (42 U/g) intraperitoneal injection every other day to support the survival and proliferation of the NKTL cells. To detect the effect of the Chiauranib in vivo, mice injected with shNC-SNK6 or shAIF-SNK6 cells were randomized and divided into two groups, respectively (*n* = 5 in each group). Mice received Chiauranib or DMSO treatment (0.18 μmol/g, intraperitoneal injection, every other day) 1 week after inoculation with NKTL cells. Three weeks after the inoculation of NKTL cells, mice were euthanized. Exclusion criteria: Mice that fail to develop tumors will be excluded from the experimental group. Tumor tissues were dissected, weighed, taken photos, and stored at −80°C for further experiments.

For in vivo synergistic effect experiment, twenty 4-week-old NOD/SCID male mice (16–20 g) injected with SNK6 cells were randomized and divided into four groups respectively (*n* = 4 in each group). Mice received DMSO, 330 IU/kg of L-asparaginase (intraperitoneal injection, every other day), 0.18 μmol/g Chiauranib (intraperitoneal injection, every other day), or a combination of both within 2 weeks after inoculation with SNK6 cells. Four weeks after the inoculation of NKTL cells, mice were euthanized. Exclusion criteria: Mice that fail to develop tumors will be excluded from the experimental group. All experiment procedures were reviewed and approved by the Institutional Animal Care and Use Committee of Sun Yat-sen University.

### siRNA, plasmids, lentivirus, and transfection

Lentiviral vectors contained the puromycin resistance gene. Vectors encoding AIF shRNA were purchased from GeneChem Co., Ltd. (Shanghai, China). VDAC siRNA was purchased from RiboBio (Guangzhou, China). Plasmids encoding Bax were purchased from Obio Technology (Shanghai, China) Corp, Ltd; Cells were transduced with packaged lentivirus and subjected to puromycin selection as previously described. According to the manufacturer’s instructions, transfections were performed at approximately 50% confluency using RNAiMAX (Invitrogen, USA). After 48 h, confirmation of interference was carried out using real-time quantitative PCR (RT-qPCR) and Western blotting.

### RNA isolation and RT-qPCR

Total RNA was extracted from NKTL cell lines according to the manufacturer’s instructions for the kit (Tiangen, Beijing, China). Then, 500 ng total RNA was used for reverse transcription using PrimeScript1RT reagent Kit Perfect Real Time kit (Takara Bio, Inc., Shiga, Japan) and then subjected to quantitative real-time PCR analysis (qPCR) using iQ SYBR Green Supermix and the iCycler Real-time PCR Detection System (Bio-Rad, USA). Relative mRNA quantities were determined using the comparative cycle threshold (2−∆∆Ct) method. β-actin was used for normalization. Primer sets for SYBR Green analysis of human AIF, Bax, VDAC, MGA, MYC, EP300, MEF2C, PD-L1, PD-L2, and β-actin are as follows:

AIF (Forward primer: 5’- AAGTCAGACGAGAGGGGGTTA-3’; Reverse primer: 5’- GCCAACTCAACATTGGGCT-3’),

Bax (Forward primer: 5’- CCCGAGAGGTCTTTTTCCGAG-3’; Reverse primer: 5’- CCAGCCCATGATGGTTCTGAT’);

VDAC (Forward primer: 5’- ACGTATGCCGATCTTGGCAAA-3’; Reverse primer: 5’- TCAGGCCGTACTCAGTCCATC’);

β-actin (Forward primer: 5’- ACTCTTCCAGCCTTCCTTC-3’; Reverse primer 5’- ATCTCCTTCTGCATCCTGTC -3’);

MGA (Forward primer: 5’- TAAGCCTCAAGTTGCCGGTA -3’; Reverse primer: 5’- CAGATGGTCAGATGGCTTGC -3’),

MYC (Forward primer: 5’- GGCTCCTGGCAAAAGGTCA-3’; Reverse primer: 5’- CTGCGTAGTTGTGCTGATGT-3’),

EP300 (Forward primer: 5’- ACTTGGAGCACGACTTACCA-3’; Reverse primer: 5’- CCCATGGCAGGCTGATTTAC-3’),

MEF2C (Forward primer: 5’- GGTATGGCAATCCCCGAAAC-3’; Reverse primer: 5’- CTCCCATTCCTTGTCCTGGT-3’),

PD-L1 (Forward primer: 5’- CACGGTTCCCAAGGACCTAT-3’; Reverse primer: 5’- CTTGGAATTGGTGGTGGTGG-3’),

PD-L2 (Forward primer: 5’- CCAACTTGGCTGCTTCACAT-3’; Reverse primer: 5’- AGTCTGGCAGCAAGAAGGAT-3’)

### Western blotting

The total protein was collected using SDS lysis buffer (Beyotime, P0013G), and protein concentration was determined by Bicinchoninic Acid (BCA) method according to the manufacturer’s protocol (KeyGen, KGP902). Nucleus extracts were obtained using the NE-PER Nucleus and cytoplasmic extraction reagents (Thermo, Rockford, AL, USA) according to the manufacturer’s instructions. Mitochondria fractions were performed using a Mitochondria Isolation Kit (APPLYGEN, Beijing, China) according to the manufacturer’s instructions. Proteins were separated by SDS-PAGE and transferred to 0.45 μm PVDF membranes (Millipore) according to standard immunoblotting protocols. After blocking with 5% of defatted milk in TBST (20 mM of Tris-HCl pH 7.4, 500 mM of NaCl and 0.1% of Tween-20) for 1 h at room temperature, the membranes were incubated with the following primary antibodies: p-Akt (T308) (#13038), Akt (#4691), p-GSK3β (T308) (#9323), GSK3β (#9832), AIF(#4642), Caspase-8 (#9746), Caspase-9 (#9502), Histone (4499) from Cell Signaling Technology; Cleave-caspase-8 (AC033), m-Calpain (AC012) from Beyotime Co., Ltd.; COX-IV (ab14744) from Abcam; β-actin (A5441) from Sigma-Aldrich; Cytochrome c (sc-13156), VDAC (sc-390996), Bax (sc-6236) from SantaCruz. After incubation at 4 °C overnight, membranes were probed with HRP-conjugated anti-rabbit IgG (Cell Signaling Tech, #7074) or anti-mouse IgG (Sigma-Aldrich, AP308P), then developed by ECL substrate (Merck Millipore) and visualized using the Bio-Rad ChemiDoc Touch Imaging System.

### Immunofluorescence

Immunofluorescence was performed according to a standard protocol as described previously [38]. After being fixed in 4% paraformaldehyde, cells were blocked with goat serum at 37 °C for 1 h. They were incubated with rabbit AIF antibodies at 37 °C for 2 h, then were incubated with FITC conjugated goat anti-rabbit IgG (Dako, Glostrup, Denmark) at 37 °C for 1 h after three times washing. Finally, the cell nucleus was stained with DAPI (Sigma-Aldrich). Cells were visualized under Olympus BX51.

### Immunohistochemistry

Immunohistochemistry was performed according to a standard protocol as described previously [39]. Tumor tissues were fixed with 4% paraformaldehyde and cut into 5-μm paraffin-embedded sections. After endogenous peroxidase quenching and goat serum blocking, the slides were incubated with anti-Bax monoclonal antibody at 4°C overnight. On the second day, the slides were treated with HRP-conjugated secondary antibody, and the antigen-antibody complex was visualized by incubation with the DAB kit. Finally, all sections were counterstained with hematoxylin and photographed through a slide scanner (Axio Scan. Z1, ZEISS). The stained sections were assessed at ×200 magnification, and 10 representative staining fields of view in each section were analyzed to determine the mean optical density (MOD), which represents the strength of the staining signals measured as the proportion of positive pixels.

Tumor MVD was performed by immunostaining using a rat anti-mouse CD31 endothelial marker. Tumor MVD was quantified using Weidner’s method. Briefly, the whole tumoral section was scanned at low power by microscope and identified the area of highest neovessel density, the so-called hot spot. Then, individual microvessels are counted at higher power (×200 field) in an adequate area. The MVD of the tumors was determined by counting CD31-positive areas in 10 fields/serial tumor sections from five animals per group. Any stained EC or clusters separate from adjacent vessels are counted as a single microvessel, even in the absence of a vessel lumen. Every single count is expressed as the highest number of microvessels identified at the hot spot. Negative controls were incubated without the primary antibody.

### Cell Counting Kit-8 (CCK8)

Cell proliferation was measured via cell viability with a Cell Counting Kit-8 (Dojindo, Japan). Cells were seeded into 96-well plates and cultured for 12, 24, 36, and 48 h. Then, 100 μl CCK8 reagent was added to 96-well plates and incubated for 2 h. The absorbance (OD450 nm) was measured using a microplate reader (TECAN, Switzerland) and calculated.

### Analysis of apoptosis by Annexin V/propidium iodide, Hoechst 33258, and TUNEL staining

Apoptosis was assessed by Annexin V/propidium iodide (PI), Hoechst, and TUNEL staining detection as described previously. For irreversible Annexin V/PI analysis, Pan-caspase inhibitor Z-VAD-FMK (Beyotime), Akt activator SC-79(Selleck), and GSK inhibitor LiCl (Sigma-Aldrich) were dissolved according to the manufacturer’s recommendation. Cells were pretreated with these reagents for 30 min and then co-treated with the indicated concentration of Chiauranib for another 24 h. Colchicine (Sigma-Aldrich) was performed as the positive control. Alternatively, for Hoechst staining, the cells were labeled with 5 μg/ml of Hoechst 33258 for 20 min at 37°C and examined by fluorescence microscopy (Olympus BX51, Olympus).

### Calcium measurement

Calcium measurement kit (Yeasen, catalog number: 40776ES50). Cells were treated with the indicated concentration of Chiauranib for 48 h, before which the medium was exchanged with phosphate-buffered saline (PBS) containing 2 μM Rhod-2 AM and the indicated concentration of Chiauranib, incubated for another 2 h. After the cells were washed three times with PBS, results were obtained using a Beckman Coulter flow cytometry.

### Calpain activity measurement

Calpain activity measurement kit (APExBio, catalog number: K2062). Cells were resuspended in 200 μl of calpain reaction buffer (50 mM Tris-HCl, 50 mM NaCl, 5 mM β-mercaptoethanol, 1 mM EDTA, 1 mM EGTA, pH 7.5, filtered by a 0.2-μm membrane) containing 0.1% Triton X-100. The cell lysate was transferred into a 96-well white plate (Corning, USA), and calpain substrate was added to each well. Fluorescence was detected at an emission wavelength of 420 nm with excitation at 320 nm and quantitated as relative fluorescence units (RFUs) using a kinetic method for 180 min at 37°C with a SpectraMax M5 Fluorescence Reader.

### Statistical analysis

Statistical analysis was performed using Prism software (GraphPad Software 8.0). Two-way ANOVA with Tukey’s multiple comparisons test was used for grouped analysis. Significant changes between the two groups were analyzed with the *t*-test. Two-sided *P*-values <0.05 were considered significant, and the level of significance was indicated as *P < 0.05, **P < 0.01, ***P < 0.001, and ****P < 0.0001. All data are presented as mean ± SD.

## Supplementary information

Supplementary figure legends

Supplementary figures

Western blot Original Data File

Checklist

## Data Availability

All study data are included in the article.
