# Supplementary material for: Inhibition of extranodal NK/T-cell lymphoma by Chiauranib through an AIF-dependent pathway and its synergy with L-asparaginase
Source: Cell Death Dis. 2023 May 9;14(5):316. doi: 10.1038/s41419-023-05833-w (PMC10169864; doi:10.1038/s41419-023-05833-w)
Supplement: Supplementary file 1 — Supplementary figure legends [file 41419_2023_5833_MOESM1_ESM.docx]

**Supplementary Figure 1**

1. Immunoblotting analysis of AIF shRNA knock down expression in SNK6 cell line. (B) Immunoblotting analysis of VDAC-1 siRNA knock down expression in SNK6 cell line. (C) Immunoblotting analysis of Bax over expression in SNK6 cell line. (D) Immunoblotting analysis of mitochondria and cytosol enriched fractions of SNK6 cells after Bax overexpression.

**Supplementary Figure 2**

Figure 1. Expression of MGA, MYC, EP300, MEF2C, PD-L1, and PD-L2 in NK-YS and SNK-6 cells. Assay was set up in triplicate. Data were represented as mean ±SD.
