# Supplementary figures and images for: Inhibition of extranodal NK/T-cell lymphoma by Chiauranib through an AIF-dependent pathway and its synergy with L-asparaginase
Source: Cell Death Dis. 2023 May 9;14(5):316. doi: 10.1038/s41419-023-05833-w (PMC10169864; doi:10.1038/s41419-023-05833-w)

**A**

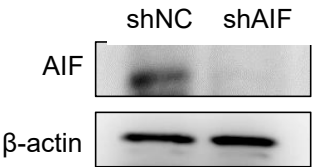

**B**

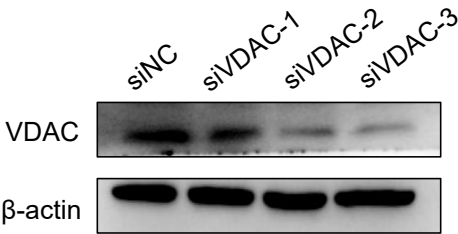

**C**

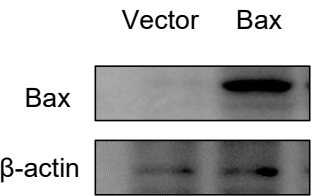

**D**

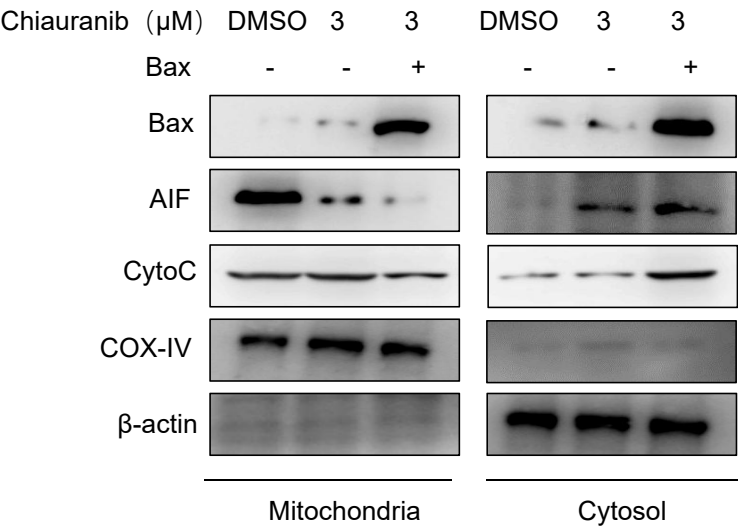

A

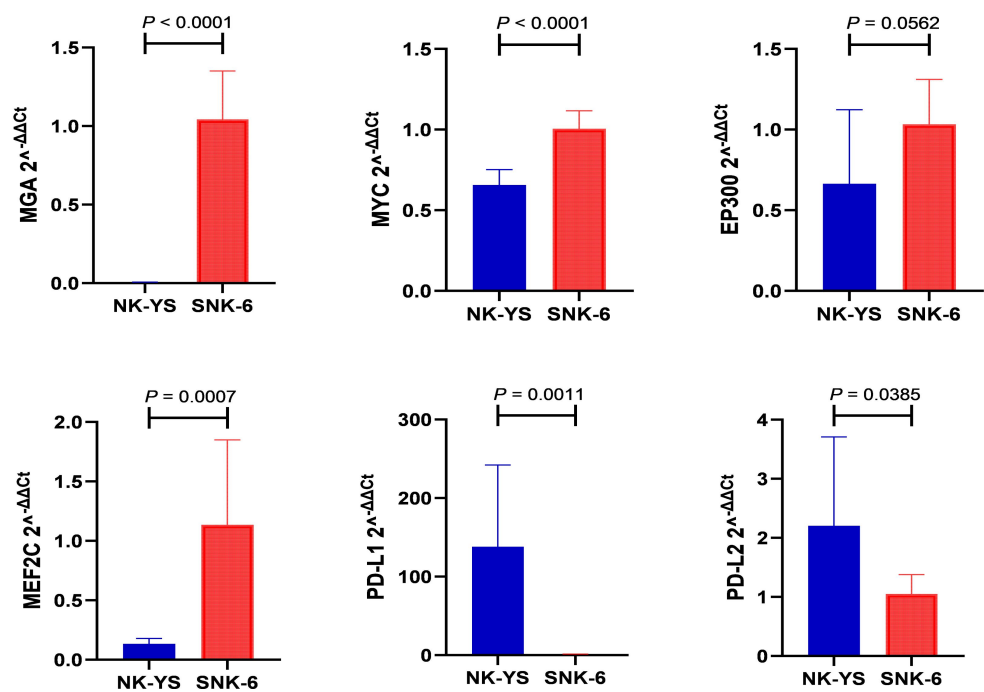

Supplement: Supplementary file 2 — Supplementary figures [file 41419_2023_5833_MOESM2_ESM.pdf]

# Figure 3

A

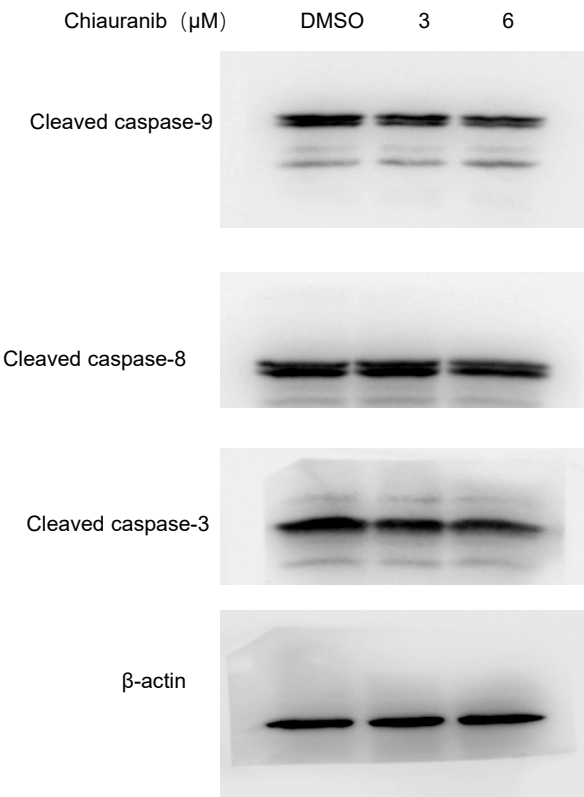

B

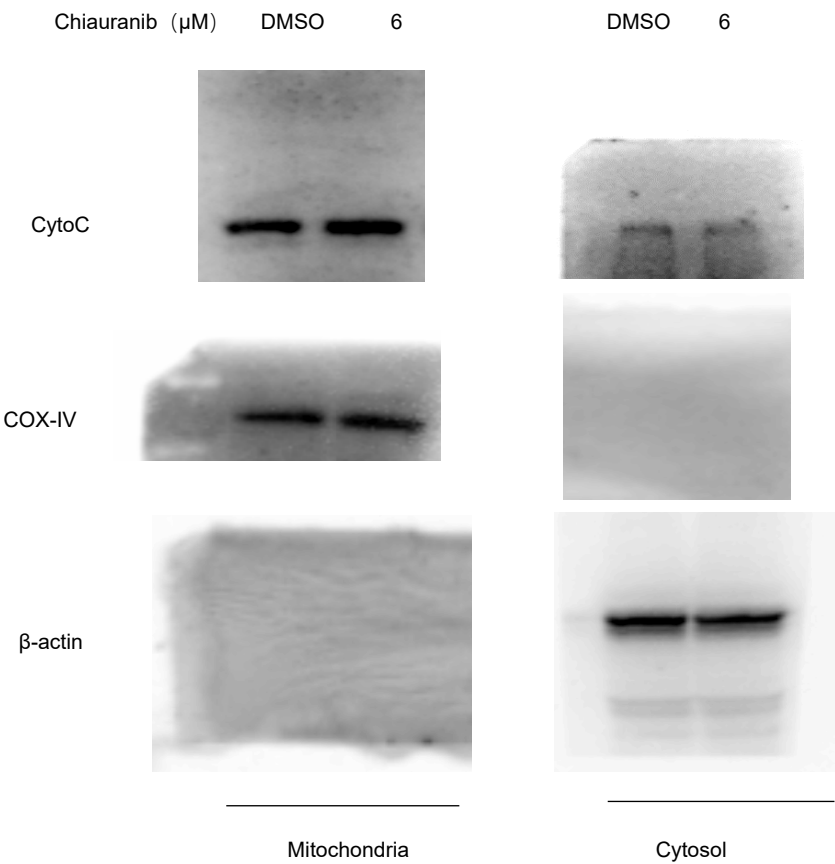

# Figure 4

C

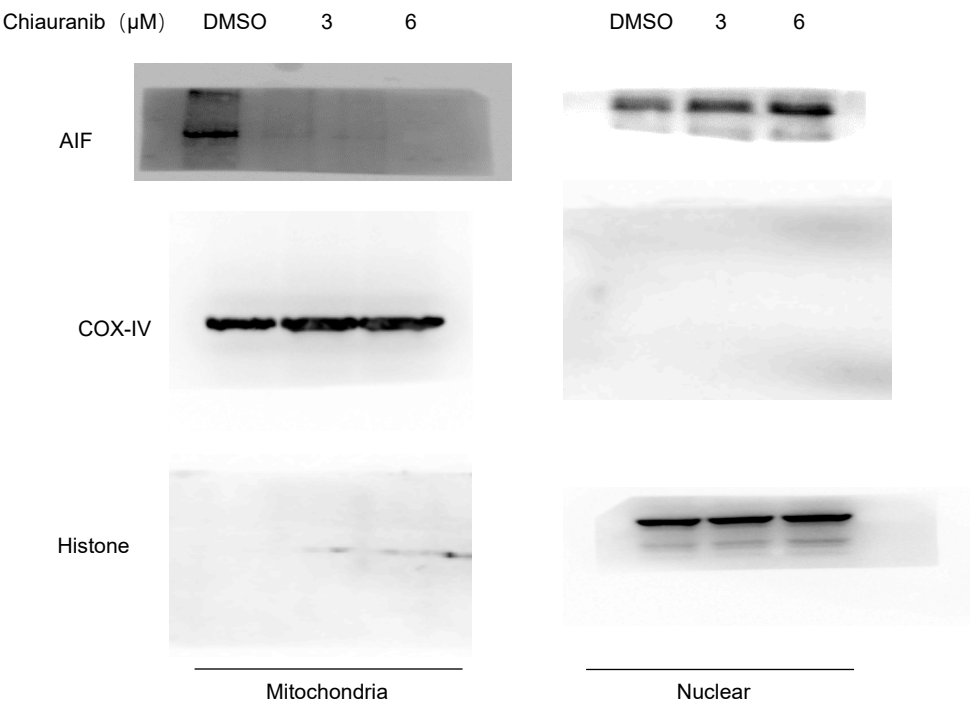

F

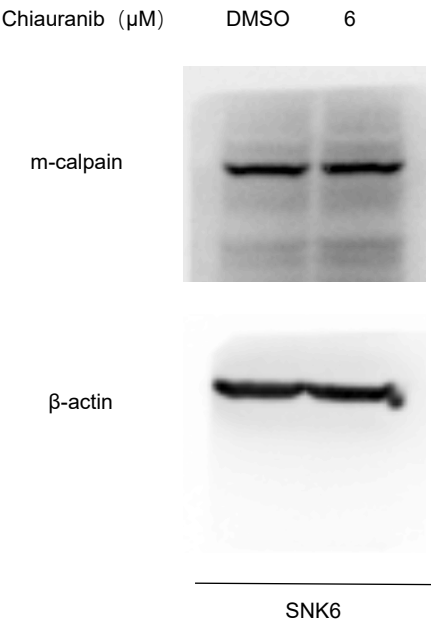

Figure 5

A

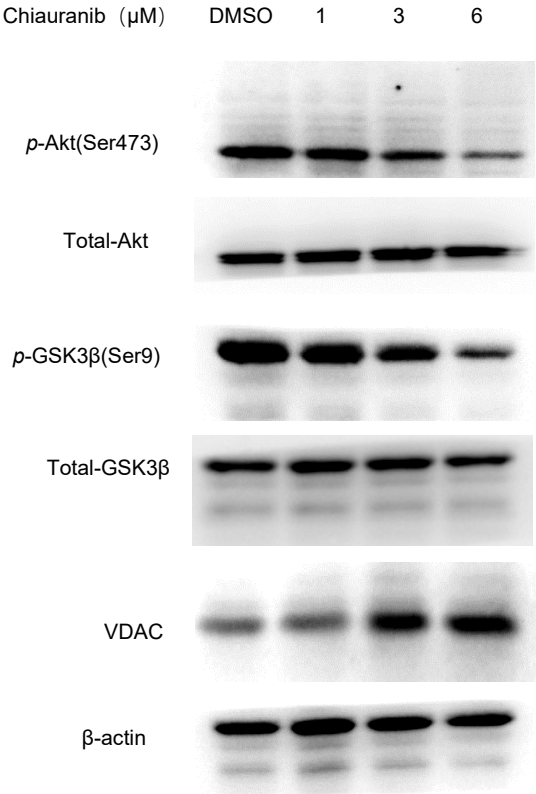

B

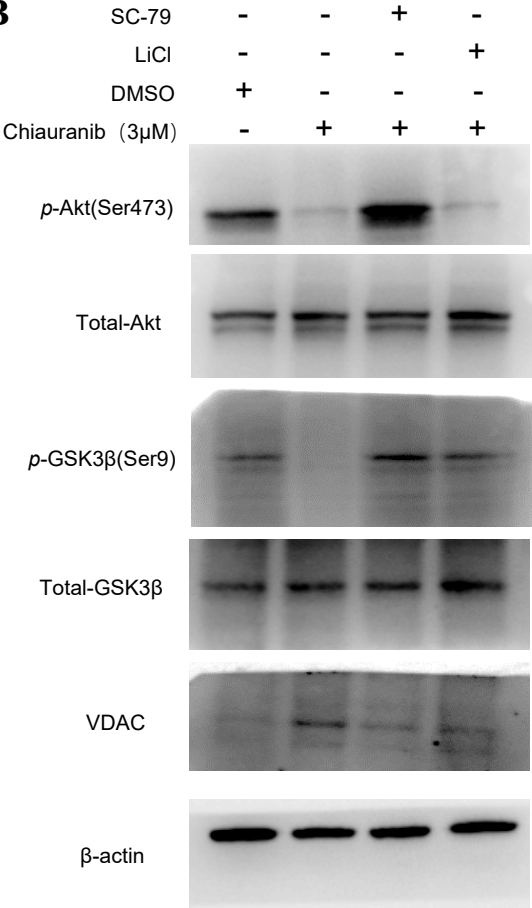

C

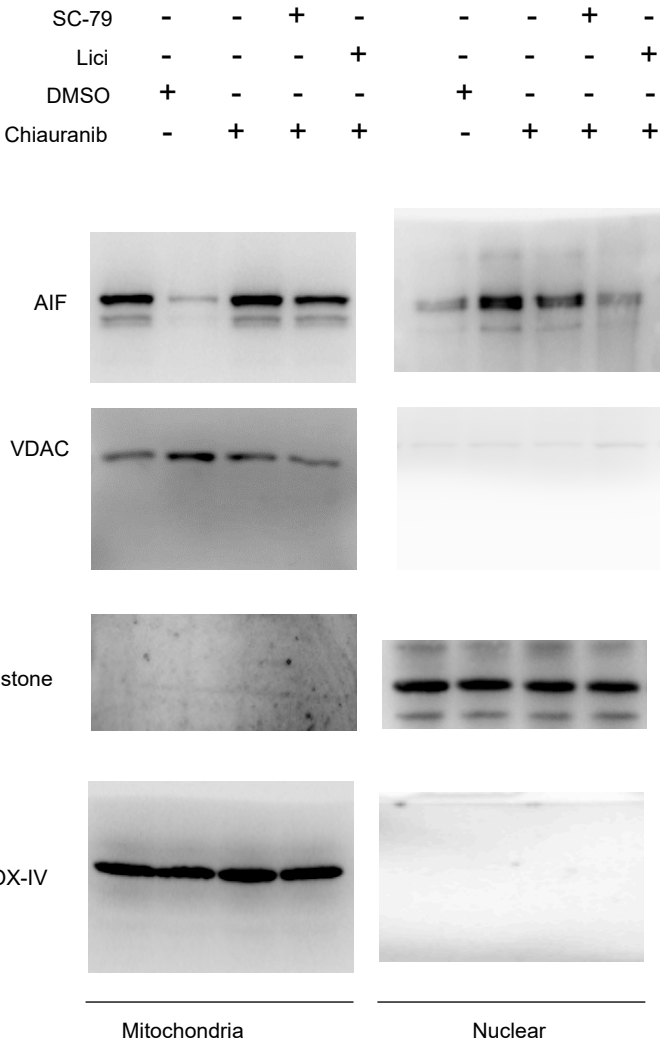

D

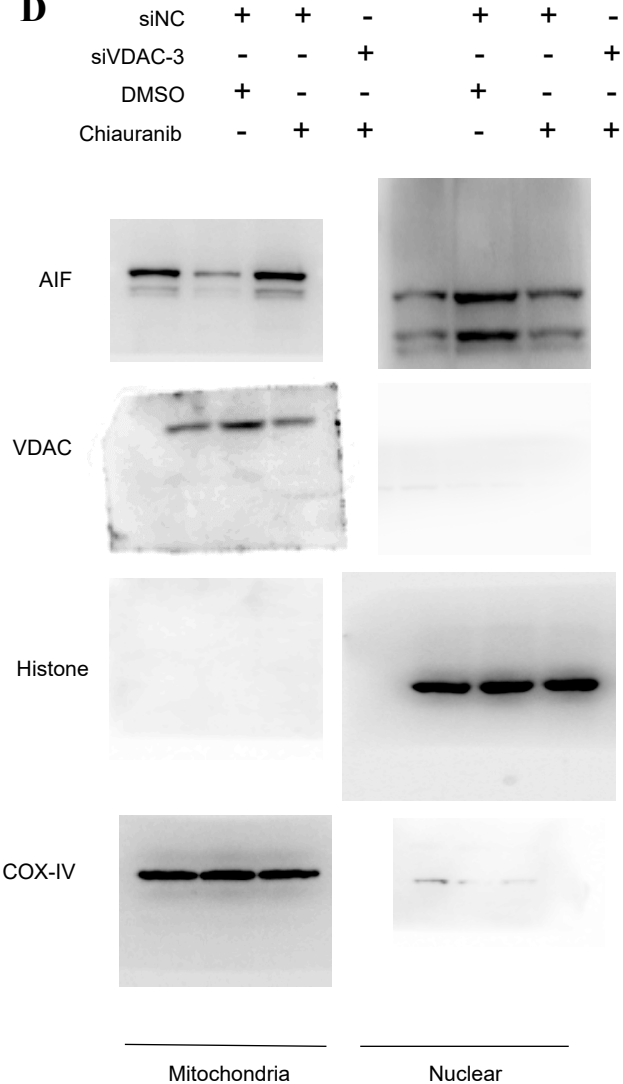

Figure 6

A

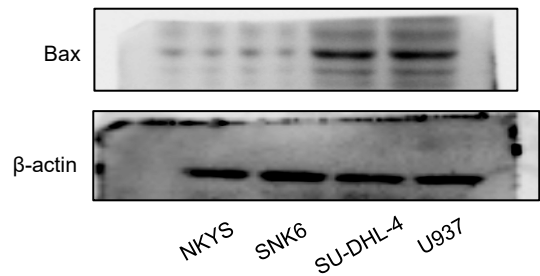

D

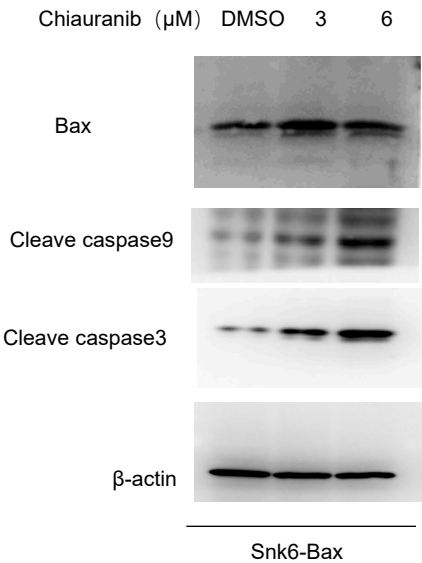

E

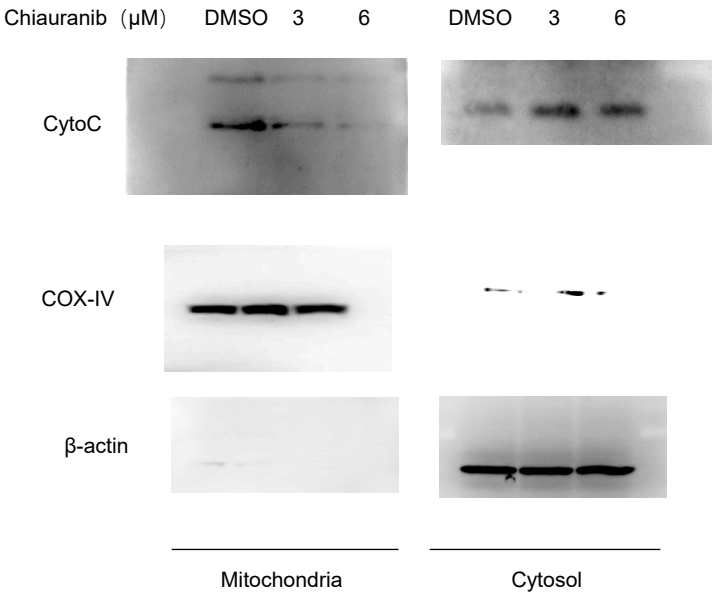

Figure 7

C

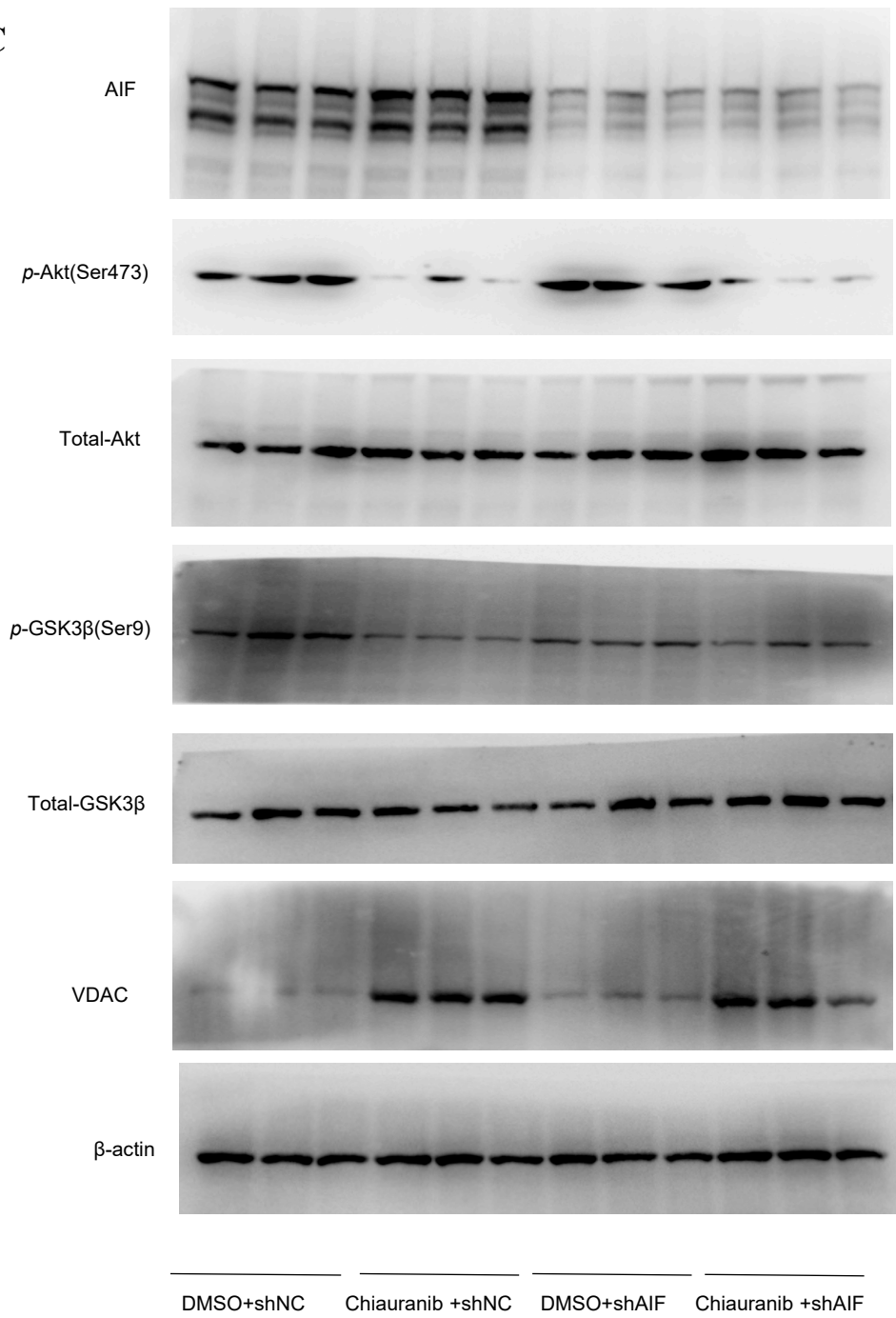

Supplement: Supplementary file 3 — Western blot Original Data File [file 41419_2023_5833_MOESM3_ESM.pdf]
